# Antioxidants Keep the Potentially Probiotic but Highly Oxygen-Sensitive Human Gut Bacterium *Faecalibacterium prausnitzii* Alive at Ambient Air

**DOI:** 10.1371/journal.pone.0096097

**Published:** 2014-05-05

**Authors:** M. Tanweer Khan, Jan Maarten van Dijl, Hermie J. M. Harmsen

**Affiliations:** Department of Medical Microbiology, University of Groningen, University Medical Center Groningen, Groningen, The Netherlands; German Institute of Human Nutrition Potsdam-Rehbrücke, Germany

## Abstract

The beneficial human gut microbe *Faecalibacterium prausnitzii* is a ‘probiotic of the future’ since it produces high amounts of butyrate and anti-inflammatory compounds. However, this bacterium is highly oxygen-senstive, making it notoriously difficult to cultivate and preserve. This has so far precluded its clinical application in the treatment of patients with inflammatory bowel diseases. The present studies were therefore aimed at developing a strategy to keep *F. prausnitzii* alive at ambient air. Our previous research showed that *F. prausnitzii* can survive in moderately oxygenized environments like the gut mucosa by transfer of electrons to oxygen. For this purpose, the bacterium exploits extracellular antioxidants, such as riboflavin and cysteine, that are abundantly present in the gut. We therefore tested to what extent these antioxidants can sustain the viability of *F. prausnitzii* at ambient air. The present results show that cysteine can facilitate the survival of *F. prausnitzii* upon exposure to air, and that this effect is significantly enhanced the by addition of riboflavin and the cryoprotectant inulin. The highly oxygen-sensitive gut bacterium *F. prausnitzii* can be kept alive at ambient air for 24 h when formulated with the antioxidants cysteine and riboflavin plus the cryoprotectant inulin. Improved formulations were obtained by addition of the bulking agents corn starch and wheat bran. Our present findings pave the way towards the biomedical exploitation of *F. prausnitzii* in redox-based therapeutics for treatment of dysbiosis-related inflammatory disorders of the human gut.

## Introduction

Diabetes, obesity, inflammatory bowel diseases and various other metabolic disorders are intimately linked to changes in the human gut microbiota [1]–[4]. It is thus important to develop strategies and tools to revert the condition of ‘dysbiosis’, where the human gut microbiota is substantially altered, to situations as typically encountered in healthy individuals [1], [5]–[9]. Therefore, the so-called ‘probiotic’ supplementation of gut microorganisms is being considered as a highly promising novel therapeutic approach for treating dysbiosis [7], [10], [11]. Such probiotics are historically defined as ‘live microbial food supplements that have beneficial effects on the host by improving its intestinal microbial balance’ [12]. Importantly, the probiotics required for treating dysbiosis do not only have to compete with pathogenic bacteria and suppress them, but they should also help to positively modulate the gut-microbial fermentation pathways [13]. This calls for the development of novel probiotic formulations that include microorganisms from the human gut microbiome, a formidable resource of beneficial microbes.

Traditionally, the bacteria *Lactobacillus acidophilus, Lactobacillus casei, Bifidobacterium bifidum* and *Bifidobacterium longum*, and the yeast *Saccharomyces boulardii* have been employed as probiotics in humans. These microorganisms are generally well-accepted and tolerated amongst consumers [13]. Alternatively, the growth and/or activity of particular gut microbes can be stimulated with non-digestible food constituents, such as inulin-type fructans [14]. Such food supplements are often referred to as ‘prebiotics’ [11], [15]. Generally, prebiotic supplementations are aimed to modulate the composition of the gut microbiota in such a way that the potentially health-promoting bacteria, such as lactobacilli and bifidobacteria are stimulated [15], [16]. ‘Synbiotics’ are combinations of probiotics and prebiotics that have a beneficial impact on the host by improving the survival and establishment of beneficial microbes in the gastrointestinal tract [11], [17]. Such synbiotics may selectively stimulate microbial growth by activating the metabolism of one, or a limited number of health-promoting microbes. Importantly, the ingestion of probiotics, prebiotics or synbiotics can modulate the short-chain fatty acid (SCFA) profiles in the human colon [11]. Here, the most abundant SCFAs are acetate, propionate and butyrate, which are generally detectable in human feces at ratios of 60∶20∶20, respectively [18], [19]. The butyrate level is of particular importance, because this SCFA represents the most preferred energy source for colonocytes [19],[20], stimulates cell proliferation [21], and promotes mucus secretion from colonic mucosa [22]. In view of these benefits of butyrate, butyrogenic bacteria are highly promising future probiotics.

One of the most important butyrate-producing bacteria in the human colon is *Faecalibacterium prausnitzii*. This bacterium and its close relatives, which are amongst the most abundant gut bacteria, belong to the clostridial cluster IV [23]. Notably, faecalibacteria are present at low numbers in patients with inflammatory-bowel disease while, on the other hand, they display significant anti-inflammatory effects in mouse colitis models [24]. These observations imply that faecalibacteria contribute substantially to gut health, which makes them prime candidates for inclusion in probiotic or synbiotic formulations [25]. However, in contrast to the fairly oxygen-tolerant probiotics that are commercialized today, these probiotics of the future are highly oxygen-sensitive. In fact, faecalibacteria cannot even withstand a few minutes of exposure to ambient air [26]. This extreme oxygen-sensitivity is a huge challenge for the development of probiotic or synbiotic formulations that include *F. prausnitzii* or its close relatives.

Freeze-drying or lyophilization is commonly used to preserve microorganisms since lyophylates can be stored for decades [27]. Accordingly, many microbes are stored as lyophilates in important strain collections like the American Type Culture Collection (ATCC) and the National Collection of Type Cultures (NCTC). To overcome the potential viability loss during formulation, it is generally recommended to lyophilize concentrated cultures containing >10^7^ cells [27]–. This ensures the presence of sufficient viable cells after lyophilization, long-term storage and reconstitution [27], [29]. For strain preservation purposes, the survival of ∼0.1% of the original cell population is in principle sufficient [29]. In contrast, for probiotic fomulation it is important that as many bacteria as possible survive lyophilization and subsequent storage. Therefore, glycerol, mannitol, sorbitol, inulin, dextrin and Crystalean have been frequently applied as cryopreservants to protect probiotic formulations during lyophilization and to enhance their shelf-life [30], [31]. However, the selection of appropriate and compatible cryopreservants remains a major challenge and no suitable protectants were so far reported for faecalibacteria.

Inulin-type fructans are generally well-known prebiotics, which have been shown to increase the number of bifidobacteria both *in vitro* and *in vivo* [14], [32]. In addition, inulin has been widely employed as a cryopreservative in lyophilization procedures [27], [30], [31], [33]. Similarly, starch and wheat bran can serve as prebiotics [34] that possibly enhance the efficacy of synbiotic formulations [35], [36]. Antioxidants such as glutathione, ascorbate and cysteine can further enhance the viability of probiotics during storage [37]. Recently, we have shown that *F. prausnitzii* can exploit flavins and oxidized thiols as redox mediators to shuttle electrons to oxygen [38]. In this way, *F. prausnitzii* can survive and thrive under moderately oxygenized conditions as encountered in the human gut, where oxygen diffuses in from the epithelial cell layers. In the present study, we therefore investigated whether and how riboflavin and cysteine can be used to develop formulations that preserve viable *F. prausnitzii* cells under oxygenized conditions. Our findings show that this is indeed a feasible strategy, which paves the way for the development of synbiotics containing faecalibacteria.

## Materials and Methods

### Bacterial strains and culturing conditions

*F. prausnitzii* strain A2-165 (DSM 17677), previously described [26] was maintained at 37°C on yeast extract, casitone, fatty acid and glucose medium (YCFAG) in an anaerobic chamber. To maintain anaerobic conditions a gas mixture of N2 (81%), H2 (11%) and CO2 (8%) was used. For formulation experiments, the bacterial cells were grown in 50 ml of YCFAG broth to an optical density at 600 nm of ∼1. The average yield of bacterial cells on a wet-weight basis was around 0.1 g per 50 ml of broth. Cells were harvested by centrifugation (3300 g) for 10 min. The resulting pellet was then washed in PBS and re-centrifuged to obtain a cell pellet that was used for further experiments.

### Formulation procedure

Bacterial cell pellets from 50 ml cultures were re-suspended in 400 µl of inulin solution (10%) with or without 0.2% cysteine or phosphate-buffered saline (PBS). To these mixtures was added either 200 µl of a 16.5 mM riboflavin solution in PBS, or 200 µl PBS without further additions. In some experiments, the bacterial slurry was later mixed with 0.5 g of wheat bran, with or without 1 g of corn starch. The mixtures were then homogenized with a sterile spatula and frozen at −20°C. Upon freezing, the mixtures were lyophilized for 3 h and stored at −20°C until further analysis. Notably, the culturing of bacteria, harvesting, granulation, and freezing were conducted anaerobically, while freeze-drying and storage were performed aerobically.

### Stability and viability testing

For stability analyses, freeze-dried granules were exposed to atmospheric air at ambient temperatures for 0 h, 6 h, 11 h or 24 h. Next, the granules were placed in an anaerobic chamber, and rehydrated in PBS to yield 1∶10 dilutions. After rehydration, 100 µl or 200 µl of the diluent was plated on YCFAG agar and incubated anaerobically at 37°C for 24 to 36 h. Colony forming units (CFU) per ml were estimated by counting individual colonies formed on the agar plates. The relative stability of lyophilized mixtures was calculated by dividing the CFU/ml determined after exposure to ambient air for a particular period of time by the CFU/ml of the rehydrated mixture immediately after formulation (T_0_). All experiments have been repeated at least twice on different dates.

### Scanning electron microscopy

Lyophilized granules of different composition were mounted on aluminum stubs, sputtered with gold particles and examined with a JEOL 6301F scanning Electron Microscope (EM), as previously described [39].

## Results and Discussion

**Figure 1:**
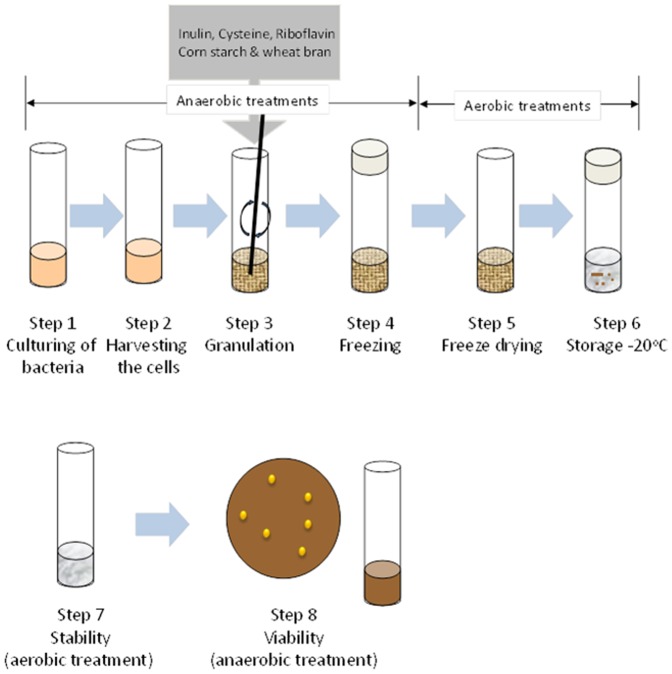
Schematic representation of the methodology employed to investigate the survival of *F. prausnitzii* in different formulations upon exposure to air. Steps 1–4 were conducted in an anaerobic chamber, and steps 5–7 were conducted aerobically in ambient air. The final step 8, involving rehydration and viability assays, was conducted in an anaerobic chamber.

**Figure 2:**
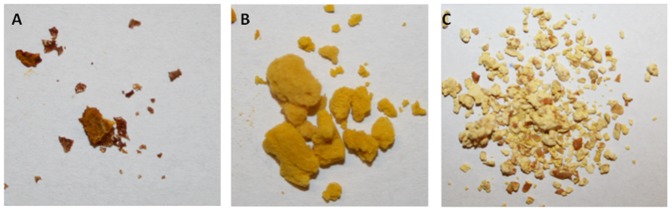
Physical appearance of freeze-dried granules containing *F. praunitzii*. A, Formulation with cysteine and riboflavin results in compact and pellet-like granules. B, Formulation with inulin, riboflavin and cysteine results in a foam-like matrix with a high bulk volume. C, Formulation with wheat bran, inulin, corn starch, cysteine and riboflavin results in hard and compact granules with considerable bulk volume.

To investigate whether it is possible to formulate *F. prausnitzii* cells in such a way that they can withstand ambient air for several hours, a formulation protocol was established as schematically depicted in Figure 1. The initial formulation steps including bacterial cultivation, harvesting and mixing with particular compounds were carried out under anaerobic conditions. After lyophilization, the resulting granules were frozen (−20°C) and stored aerobically at −20°C until further use. Depending on their composition, the lyophilized mixtures were recovered as granules with different morphologies. For example, Figure 2A shows the compact flake-like appearance of a lyophilized preparation containing cysteine, riboflavin and *F. prausnitzii* cells; Figure 2B shows the granules that were obtained when inulin was present in addition to cysteine, riboflavin and *F. prausnitzii* cells; and Figure 2C shows the hard and compact granules that were obtained when *F. prausnitzii* was formulated with corn starch, wheat bran, inulin, cysteine and riboflavin. In the latter type of formulation, the bacterial cells were found to adhere to the wheat bran and/or cornstarch. Table 1 shows the relative viability of *F. prausnitzii* in the different tested formulations upon exposure to ambient air for 6, 11 or 24 h as compared to the bacterial counts obtained immediately after the formulation (T_0_). Notably, the established cryopreservant inulin did not detectably enhance faecalibacterial survival by itself. The same was true for riboflavin, despite its known antioxidant activity. On the other hand, cysteine had a mildly protective effect, leading to about 10% bacterial survival upon 24 h exposure to air. When combined, inulin and riboflavin gave no detectable protection, but inulin and cysteine were highly effective leading to almost 60% bacterial survival. Intriguingly, the same level of protection was achieved with a combination of riboflavin and cysteine. The highest level of protection of around 70% was achieved when cysteine was combined with riboflavin and inulin (Table 1). It should be noted that the foamy material that was obtained when the faecalibacteria were formulated with cysteine, riboflavin and inulin is difficult to handle (Fig. 2B). Therefore, we tested the possibility to use corn starch and wheat bran as bulking agents. Notably, these two compounds had no protective effects on the faecalibacteria, neither by themselves nor in combination with inulin (Table 1 and data not shown). However, close to maximum faecalibacterial survival (∼60%) was observed when corn starch and wheat bran were combined with inulin, riboflavin and cysteine. This finding is important, because the respective mixture yields hard and compact granules with a considerable bulk volume (Fig. 2C). Importantly, also in the mixture containing inulin, riboflavin, cysteine, corn starch and wheat bran, the inclusion of cysteine is crucial for bacterial protection against air (Table 1). Somewhat unexpectedly, when corn starch was present, also inulin and wheat bran did contribute to the faecalibacterial survival, but a mix of only these two compounds did not support faecalibacterial survival at ambient air (Table 1). Altogether, we conclude that a formulation of *F. prausnitzii* with inulin, riboflavin, cysteine, corn starch and wheat bran is optimal both in terms of faecalibacterial survival and the type of granules that are obtained.

**Table 1:** Survival of *F. prausnitzii* in different formulations.

Combinations						Survival percentage (+/−5%)		
						Exposure to ambient air (h)		
						6	11	24
1	Inu					nd	nd	0
2		Rb				nd	nd	0
3			Cys			nd	nd	10
4	Inu	Rb				nd	nd	0
5	Inu		Cys			nd	nd	59
6		Rb	Cys			nd	nd	60
7	Inu	Rb	Cys			nd	nd	70
8				Cs		0	0	0
9				Cs	Wb	0	0	0
10	Inu			Cs	Wb	6	0	0
11	Inu	Rb	Cys	Cs	Wb	93	100	57
12	Inu	Rb		Cs	Wb	75	3	0
13		Rb	Cys	Cs	Wb	nd	45	0
14	Inu	Rb	Cys	Cs		nd	40	22
15		Rb	Cys	Cs		nd	53	0
16	Inu				Wb	nd	nd	0

Inu, Inulin; Rb, Riboflavin; Cys, Cysteine; Cs, Corn starch; Wb, Wheat bran; nd, not determined.

Please note that the data shown in Table 1 result from a single experiment. In this experiment we focused predominantly on 24 h survival at ambient air, because this period of time is probably needed for future formulation processes.

**Figure 3:**
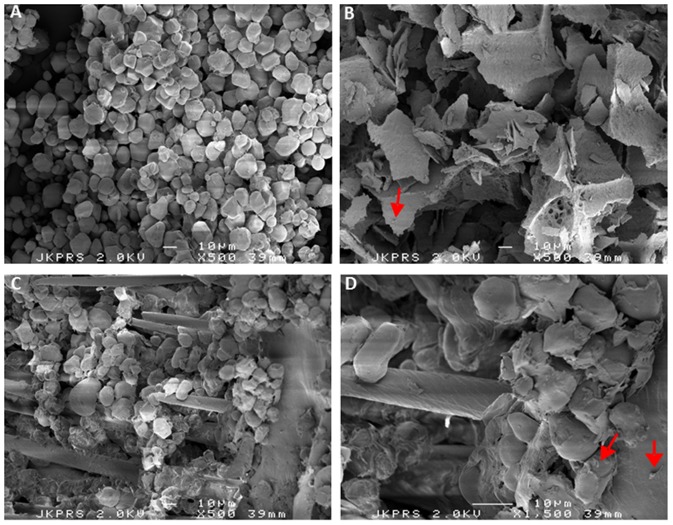
Scanning electron micrographs of *F. prausnitzii*-containing formulations. All formulations shown contained cysteine and riboflavin supplemented either with corn starch (A), inulin (B), or wheat bran, corn starch and inulin (C and D). Corn starch has a discrete bead-like appearence (A). In contrast, inulin forms a flake-like matrix (B), that can form a coating around corn starch and/or wheat bran, thereby entrapping the bacterial cells (C and D). Images A, B and C were recorded at 500× magnification, and image D was recorded at 1500× magnification. The arrows indicate entrapped *F. prausnitzii* cells.

**Figure 4:**
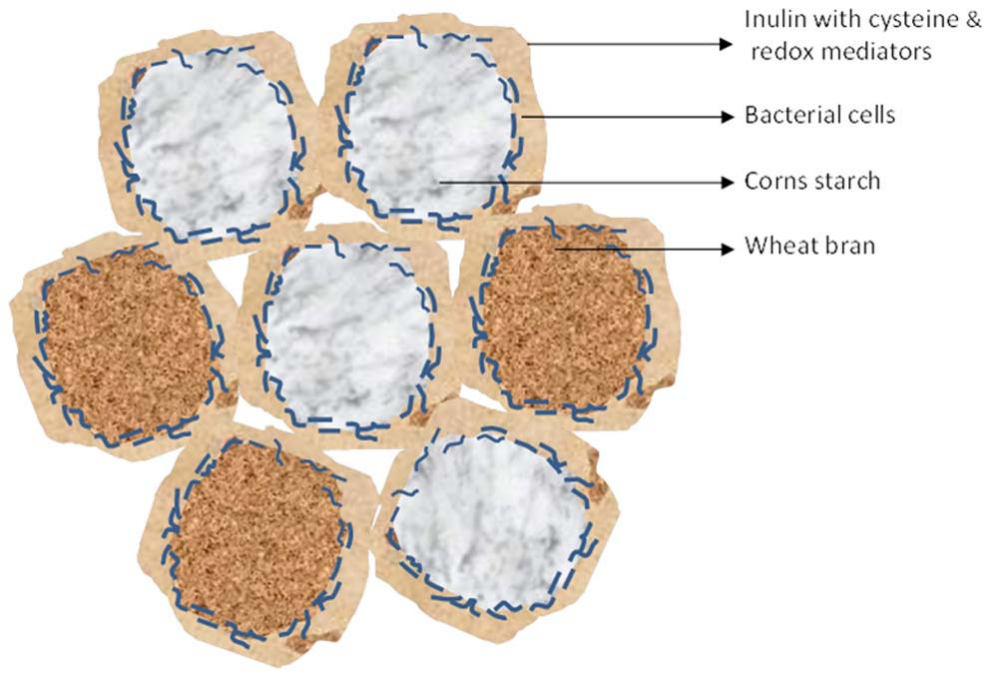
Tentative schematic representation of a formulation in which *F. prausnitzii* cells were adhered on cornstarch or wheat bran, and entrapped by an inulin matrix containing riboflavin and cysteine as antioxidants and redox mediators.

As shown by scanning EM, corn starch forms discrete bead like structures during our formulation procedure (Fig. 3A) while inulin forms a flake-like matrix (Fig. 3B). When combined, corn starch, wheat bran and inulin form a matrix upon lyophilization that seems to facilitate faecalibacterial entrapment on corn starch and wheat bran particles as shown in Figure 3 (C and D) and schematically represented in Figure 4.Keeping highly oxygen-sensitive bacteria, such as *F. prausnitzii*, alive in probiotic or synbiotic formulations has been considered a major challenge [27]. In this study, we describe a methodology for formulating *F. prausnitzii*, which allows cells of this bacterium to survive the exposure to ambient air for at least 24 h. The principal concept underlying our novel formulation is that the faecalibacterial cells are coated with antioxidants, which protects them from the lethal effects of oxygen when exposed to ambient air (Fig. 4). Bulking agents, such as wheat bran and corn starch provide a substratum for microbial cell adherence and inulin serves as a matrix to capture the faecalibacterial cells on the corn starch and wheat bran particles (Fig. 3D). Furthermore, the inulin can form an outer matrix that incorporates the antioxidants cysteine and riboflavin (Fig. 3, C and D). Notably, these compounds will not only protect the faecalibacteria from oxidative damage, but they will also facilitate faecalibacterial growth upon rehydration. The latter relates to the fact that *F. prausnitzii* can use riboflavin and oxidized cysteine to shuttle electrons to oxygen, which allows them to grow to higher cell densities in moderately oxygenized environments [38]. Inulin is well known for its cryopreservative properties [27], [30], [33] and our data suggest that it protects the faecalibacteria during lyophylization. However, inulin is not protective by itself and it can only sustain the viability of faecalibacteria in combination with cysteine or, even better, cysteine plus riboflavin. Furthermore, the addition of inulin also increases the bulk volume of the formulation (Fig. 2B). While the different bulking agents used in the present study result in variable granule sizes as shown in Figure 2, we do not believe that this will result in major differences in the surface areas exposed to oxygen since the different granules are porous and exposed to oxygen on a microscale regardless their macro size (Fig. 3). Instead, our present findings imply that the oxygen protection is provided mainly through the coating with antioxidants and inulin, which creates a reducing environment in which F. prausnitzii can survive. At this stage we do not exactly know why the survival of F. prausnitzii in the presence of bulking agents is somewhat lower than when these bulking agents are absent, but it could be due to the fact that the bulking agents wheat bran and corn starch quickly attract moisture that could lead to decreased survival upon exposure to ambient air.Notably, the bulking agents corn starch, wheat bran and inulin used in this study do not only facilitate the formulation procedure, but they are also known to confer prebiotic effects when ingested by the host [14], [34], [36]. For instance, prebiotic administration of inulin is known to result in increased numbers of *F. prausnitzii* and other beneficial gut microbes [14]. Wheat bran is a rich source of arabinoxylan, which has been suggested to lower low-grade chronic or systemic inflammation in obese mouse models [34]. In addition, food supplementation with arabinoxylan is known to counteract the dysbiosis induced by high fat diets [35]. Corn starch or resistant starch can also modulate the gut microbiota, leading to altered SCFA production profiles [40]–[43]. Lastly, cysteine is known to counteract oxidative stress at the gut mucosal lining [44], while *F. prausnitzii* is believed to have significant anti-inflammatory effects [24], [45].

Altogether, the beneficial properties of the butyrogenic bacterium *F. prausnitzii* and the compounds used for its formulation indicate that our present approach has a strong potential to deliver a novel and highly potent synbiotic. This synbiotic formulation could be beneficial for the treatment of the patients with colitis, a disease characterized by severe inflammation of the colon as well as low counts of *F. prausnitzii*. Here, it would be clearly beneficial to avail of an effective synbiotic formulation to restore high counts of *F. prausnitzii*. Our present findings represent a major step forward towards the development of such a synbiotic, as we now know how to keep the highly oxygen-sensitive bacterium *F. prausnitzii* and possibly other anaerobic gut microbes alive at ambient air.
